# Prognosis and risk factors of ERCP pancreatitis in elderly

**DOI:** 10.1038/s41598-021-95484-8

**Published:** 2021-08-05

**Authors:** Erhan Ergin, Nevin Oruç, Galip Ersöz, Oktay Tekeşin, Ömer Özütemiz

**Affiliations:** Gastroenterology Department, Faculty of Medicine, Ege University, Bornova, Izmir Turkey

**Keywords:** Biochemistry, Cancer, Biomarkers, Diseases, Gastroenterology, Health care, Medical research, Pathogenesis, Risk factors, Signs and symptoms

## Abstract

Post Endoscopic Retrograde Cholangiopancreatography (ERCP) pancreatitis is one of the most serious complications of ERCP. Our study aims to investigate the risk, predisposing factors and prognosis of pancreatitis after ERCP in elderly patients. Patients referred to the ERCP unit between April 2008 and 2012 and admitted to the hospital at least 1 day after the ERCP procedure were included to the study. Information including patient’s demographics, diagnosis, imaging findings, biochemical analysis, details of the ERCP procedure and complications were recorded. The severity of post ERCP pancreatitis (PEP) was determined by revised Atlanta Criteria as well as APACHE II and Ranson scores. A total of 2902 ERCP patients were evaluated and 988 were included to the study. Patients were divided into two groups as ≥ 65 years old (494 patients, 259 F, 235 M) and < 65 years old (494 patients, 274 F, 220 M). PEP was diagnosed in 4.3% of patients aged 65 years and older. The female gender was risk factors in elderly for PEP. The Sphincter Oddi Dysfunction (SOD) and Juxta papillary diverticula (JPD) were higher in elderly patients with PEP. Age did not increase the risk of PEP development. The most important post ERCP pancreatitis risk factor in the elderly is the female gender, while the risk is enhanced slightly by SOD and JPD.

## Introduction

Nowadays, the life expectancy of the elderly has been increasing and our population is getting older. The geriatric population is defined as ≥ 65 years old subjects. The incidence of biliary and pancreatic disorders also increase as the population ages^1^. In the United States 33% of population by the age of 70 are found to have choledocholithiasis and cholelithiasis^2^. Endoscopic retrograde cholangiopancreatography (ERCP) is an invasive procedure that has been used to diagnose and treat pancreaticobiliary diseases^3^. Since the prevalence of pancreaticobiliary disease increases with age, indications for ERCP increase in elderly. Individual decisions should be made about the ERCP since age-related diseases and co-morbidities including pulmonary and cardiac dysfunction might have impact on final outcome. The ERCP procedure related significant risk might outweigh the benefits.

According to several prospective series, the overall complication rate of ERCP or sphincterotomy, is about 5–10%^4–7^. ERCP-related complications might be grouped into specific complications of the procedure itself and nonspecific complications like side effects of sedation. One of the most serious ERCP related complications is Post ERCP pancreatitis (PEP)^8^. The five independent risk factors for PEP include precut sphincterotomy, sphincter of Oddi dysfunction (SOD), cirrhosis (patient-related factors), percutaneous-endoscopic procedure (method-related factors), and difficult cannulation 8. However, the risk of ERCP related complications is not enhanced by the advanced age, as shown by multivariate analyses^7^. In several case control studies there was no relation between old age or coexisting medical conditions and complication rate of ERCP, except liver cirrhosis^9,10^.

Our study aims to investigate the PEP incidence, risk factors and prognosis among the elderly.

## Methods

All patients referred to the Ege University Faculty of Medicine, Endoscopy Department ERCP unit between April 2008 and 2012 were evaluated. Patients admitted to the hospital at least 1 day after the ERCP procedure were included to the study. Baseline demographic characteristics, disease history and biochemical data were recorded from a computerized database. Patient demographics collected were age, gender, co-morbidities and medical history. Charleston comorbidity index was calculated for each patient. Patients who had previous history of sphincterotomy or ERCP, acute pancreatitis, cancer (biliary or pancreatic), ampullary tumors, and Billroth II gastrectomy were excluded from the study.

All procedures performed in this study involving human participants were in accordance with the ethical standards of the institutional and/or national research committee and with the 1964 Helsinki Declaration. All patients gave informed consent before ERCP. Anticoagulants or antiplatelet agents were stopped temporarily or changed before the procedure according to suggested guidelines. Before the procedure topical pharyngeal anesthesia with 10% lidocaine spray were applied. Meperidine with an induction dose of 40 to 50 mg for younger patients and 25 mg for the elderly patients and midazolam with an induction dose of 3 to 4 mg for younger patients and 2 mg for older patients were used for medication. Intestinal motility was decreased using hoscine N-butylbromide. The dose of medications was titrated in terms of the procedure duration and patient’s need by anesthesiologist. Three experienced gastrointestinal endoscopist performed all procedures. Standardized techniques were applied as patients were on the left-sided position. The standard videoduodenoscope with accessory channels of 4.2 mm diameter (Olympus, Japan) was used to perform the ERCP procedures. Automated pulse oximetry was used to continuously monitor heart rate, oxygen saturation, and blood pressure.

Beside demographic data, clinic presentation of the patient, initial diagnosis, ERCP procedures (e.g. stone extraction or precut papillotomy), ERCP diagnosis, ERCP related complications and duration of hospital stay were recorded. PEP was diagnosed if abdominal pain and elevation of serum amylase level more than threefold above the normal upper limit within 24 h after endoscopic procedure were observed^10^. Ranson’s criteria and the 2nd version of the Acute Physiologic Assessment and Chronic Health Evaluation II (APACHE II) scores were used for the classification of the pancreatitis severity. The 1991 consensus guidelines as well as Revised Atlanta criteria were applied to classify severity and complications of PEP^10^.

The chi-squared test and Student’s t-test were used whenever required for statistical comparison. Normally, distributed continuous variables are reported as mean ± standard deviation (SD). Skewed continuous variables are reported as median with interquartile ranges (IQR). *P* values below 0.05 were accepted as statistically significant. SPSS version 20.00 (SPSS Inc., Chicago, IL, USA) was used to perform all the statistical analyses. Ege University Non-Invazive Clinical Research Ethics Comittee; (Issue: 2008/02-03) approved the study protocol, and all cases included in the study provided written informed consent.

### Ethics committee approval

Approval was obtained from the Ege University Non-Invazive Clinical Research Ethics Comittee; (Issue: 2008/02-03) approved the study protocol, and all cases included in the study provided written informed consent.

## Results

**Figure 1:**
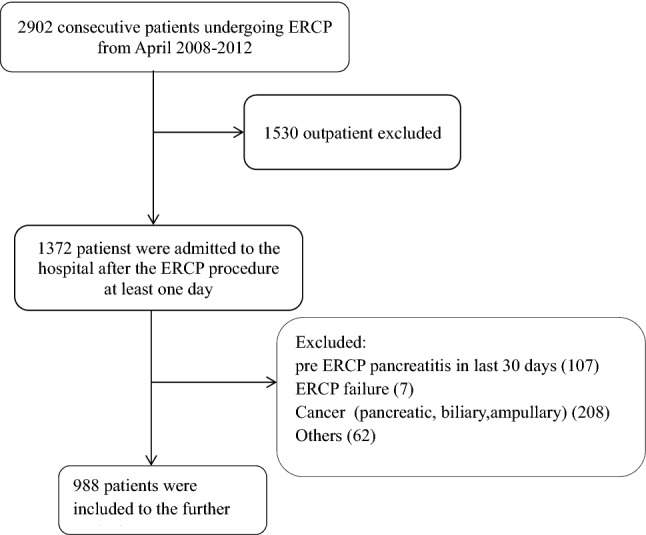
Inclusion flowchart of the study.

Between April 2008 and 2012, 2902 consecutive patients underwent ERCP procedures in endoscopy center. Patients admitted to the hospital within 1 day of the ERCP procedure (n 1372) were included in the study, while 1530 patients were excluded due to missing data. Additionally, 384 of the patients were excluded from the study due to conditions including pre ERCP pancreatitis within the last 30 days, ERCP performed to treat biliary perforation or injury, or strictures, diagnosis of cancer (pancreatic, biliary, ampullary), pancreatic disease or ERCP failure. Remaining 988 patients were included for further analysis. The patients were divided in two groups as patients younger than 65 (494 patients, 274 F, 220 M) and aged 65 and older (494 patients, 259 F, 235 M), as seen in Fig. 1.

Baseline characteristics and medical history of both groups are shown in Table 1. There were no significant differences between the two groups for gender, cardiac and coronary disease. However CMI was higher in elderly group as expected. Overall 85% patients aged 65 years and older had CMI score over 3. On the contrary 11.7% patients under 65 had CMI over 3. The difference between groups was significant (*p* < 0.001).

**Table 1:** Baseline characteristics, medical history, and ERCP complications in the group aged 65 below as compared with the elderly.

Characteristics	Group < 65 years old (n = 494)	Group ≥ 65 years old (n = 494)	*p*-value
Male /female ratio	220/274	235/259	NS
Mean age ± SD (years)	49.6 ± 14.0	76.3 ± 5.0	< 0.05
**Medical history**			
Diabetes mellitus	44 (8.9)	56 (11.3)	NS
Hypertension	41 (8.2)	93 (18.8)	NS
Coronary artery disease	22 (4.4)	52 (10.5)	NS
Cholecystectomy	90 (18.2)	115 (23.2)	NS
Cirrhosis	21 (4.2)	8 (1.6)	NS
Pulmonary diseases	12 (2.4)	36 (7.2)	NS
**Complications**			
Post-ERCP pancreatitis	21 (4.3)	21 (4.3)	NS
Bleeding	17 (3.4)	15 (3.0)	NS
Infection	1 (0.2)	1 (0.2)	NS
Perforation	1 (0.2)	3 (0.6)	NS
Death	0	0	NS

Data are given as mean ± SD or n (%).

NS: non-significant.

Successful biliary cannulation was technically achieved in 95.1% of the patients ≥ 65 years old and 96.9% of others (*p* > 0.05). According to the analysis, 21 patients (4.3%) aged 65 years and older suffered from PEP. Likewise, 21 patients (4.3%) younger than 65 years of age developed PEP (Table 1). Other rare complications related to ERCP were bleeding, infection (cholangitis), and perforation.

Patients were divided into age decades and the ratio of PEP were calculated for each decade. Patients who were 20 to 30 years old had the highest PEP risk with the 7.7% prevalence, while the patients at their sixth, seventh, eighth, and ninth decades had 4.2%, 4.7%, 4.1% and 0% pancreatitis ratios, respectively. When the prognosis of the patients was evaluated, it has been shown that the patients with post ERCP pancreatitis in the 7th decade had higher Ranson (median IQR 2 (1–3) and APACHE II scores (median IQR 6 (6–6) than those at other decades, as shown in Table 2. The indication for ERCP was choledocholitiasis in all but one at 7th decade. Two of them diagnosed with JPD. Three of patients had bleeding after ERCP and PEP. All those are possible explanations for more severe pancreatitis at 7th decade.

**Table 2:** Patients compared to see post ERCP pancreatitis ratio and prognosis and divided into their age decades.

Age decades	Patients (n)	Post ERCP pancreatitis (n, %)	Ranson score (Median, IQR)	Apache II score (Median, IQR)
11–20	1	0 (% 0)	0	0
21–30	39	3 (% 7.7)	0 (0–0)	0 (0–2)
31–40	71	5 (% 7)	1 (1–1)	0 (1–1)
41–50	104	2 (% 1.9)	2	3
51–60	184	7 (% 3.8)	1 (1–2.5)	3 (2.5–5.5)
61–70	239	10 (% 4.2)	1 (1–2)	5 (3–5)
71–80	211	10 (% 4.7)	2 (1–3)	6 (6–6)
81–90	122	5 (% 4.1)	2 (1–2)	6 (4–6)
91 years and older	17	0		

Similarly according to revised Atlanta criteria none of the elderly patients had severe PEP while only one patient under 65 years old had severe pancreatitis. In elderly group 57.9% of patients had moderate and 42.1% had mild pancreatitis while 31.6% and 63.2% respectively in patients < 65 years old. The difference between two groups was not significant. The median IQR CMI score was 2 (1–3) in patients with PEP (n 42) and 2 (1–4) in others (*p* > 0.05). The CMI score of patients with PEP aged 65 and older was median IQR 3 (3–4). There was positive correlation with CMI and APACHE II scores (R 0.404, *p* < 0.01).

Median IQR hospital stay was 3 (2–7) days in patients aged below 65 years old and 4 (2–9) in patients aged or over 65 years old. There was no significant difference for length of hospital stay between two groups. None of the patients required additional interventions and no mortality was observed in both groups.

Multivariate and univariate regression analysis of the patients to determine potential confounders such as age, sex, additional disease, drug use, previous pancreatitis history etc. for PEP were performed. Additionally, ERCP related technical risk factors such as difficult cannulation, precut, pancreatic channel cannulation, presence of JPD, number and diameters of drainage stents, papilla biopsy, hemorrhage were analyzed for all patients. Post ERCP pancreatitis risk was enhanced based on female gender by OR 8.1 (95% CI 1.7–37.7) *p* 0.001), and post ERCP pancreatitis risk was enhanced based on JPD by OR 1.1. The elderly patients with post ERCP pancreatitis experienced more prevalent ERCP diagnosis of SOD than the patients who did not have pancreatitis but the difference was not significant. (14.3% vs. 3.8% respectively, *p* 0.059).

## Discussion

This is one of the largest series that evaluates the PEP among the elderly. Although there is growing life expectancy in developed countries, the mean age in these countries is still 65 years. ERCP is an effective method used for the diagnosis and treatment of pancreatobiliary diseases^11^. The data on ERCP related complications in elderly has been reported in numerous prospective and retrospective studies (Table 3). Our study suggests similar success rate of the selective biliary cannulation and PEP between both elderly and younger groups.

**Table 3:** Reported series about ERCP in elderly.

Author year (reference no.)	Patient age (range)	ERCP (n)	Study type	Complications
MacMahon et al. (1993)^15^	65–94	50	Prospective	None
Deans (1997)^21^	≥ 65	677	Prospective	Perforation, cholangitis, pancreatitis, bleeding, deaths
Ashton (1998)^22^	75–100	101	Retrospective	Cholangitis, pancreatitis, perforation
Sugiyama (2000)^23^	> 90 vs. 70–89	403	Retrospective	Death, pancreatitis, bleeding, cholangitis, hepatic failure, basket impaction
Clarke (2001)^12^	85–94	21	Prospective	1 pancreatitis
Mitchell et al. (2003)^24^	≥ 90	23	Retrospective	bleeding
Gonzales et al. (2003)^14^	≥ 90	126	Retrospective	Deaths, cholangitis, bleeding
Garcia-Cano (2003)^25^	≥ 90	16	Retrospective	cholangitis
Hui et al. (2004)^26^	≥ 90 vs. < 90	229	Retrospective	Pancreatitis, bleeding, desaturation, hypotension, atrial fibrillation
Köklü et al. (2005)^13^	≥ 70 vs. < 70	299	Retrospective	Bleeding, pancreatitis, perforation, cholangitis, ileus, death
Fritz et al. (2006)^27^	≥ 80 vs. < 80	502	Retrospective	Bleeding, pancreatitis, perforation, cholangitis, respiratory insufficiency, bradycardia
Thomopoulos et al. (2007)^28^	≥ 80	209	Retrospective	Pancreatitis, aspiration, cholangitis, cholecystitis, bleeding, retroperitoneal perforation, esophageal perfotarion
Riphaus et al. (2008)^16^	≥ 80	1313	Prospective	Pancreatitis, bleeding
Katsinelos et al. (2011)^17^	≥ 80 vs. < 80	600	Prospective	Pancreatitis, bleeding, perforation, basket impaction, cholangitis, death
Gronroos et al. (2010)^29^	≥ 90	41	Retrospective	bleeding
Garcia et al. (2016)^30^	≥ 75 vs. 65–74	89	Retrospective	Pancreatitis, atrial fibrillation, death bleeding
Katsinelos et al. (2018)^31^	≥ 75 vs. < 75	2688	Retrospective	Pancreatitis
Maitin-Casalis et al. (2015)^32^	≥ 80 vs. 65–80	1044	Retrospective	Pancreatitis
Han et al. (2016)^33^	≥ 80 vs. 65	624	Retrospective	Pancreatitis
Tohda et al. (2016)^34^	≥ 80 vs. < 80	207	Retrospective	Bleeding, pancreatitis, cholangitis, Aspiration pneumonia
Maitin-Casalis (2015)^35^	≥ 80 vs. 50–79	1284	Prospective	Pancreatitis
Syrén et al. (2019)^36^	≥ 65 vs. < 65	15.800	Retrospective	Unspecified
Current study	≥ 65 vs. < 65	988	Retrospective	Pancreatitis, bleeding, perforation, cholangitis

Demand on ERCP as an therapeutic procedure among the elderly raised due to the increase in the malignant biliary diseases, bile duct stone prevalence and elevated risk of surgery. The center and endoscopist experience might affect the success ratio of ERCP in different series. An experienced endoscopists in our center performed all procedures, and the success ratio was not affected by the patient’s age. There was technically successful selective biliary cannulation in 95.1% of the patients aged ≥ 65 years and 96.9% of other patients in our study. Cesar J. Garcia et al. reported more technically successful selective biliary cannulation among the younger patients (94.3%) than in, the older ones (77.8%)^1^. The differences in the experience of the operator might have resulted in the conflict in the reported success rates of cannulation.

Younger patients have been shown to have an increased risk of PEP (Table 3). Unfortunately, the definition of younger age among studies varies considerably since cut-off values of 50, 60, and 70 have all been used. In this study, we preferred the age limit of 65 as the most accepted definition of the geriatric population. According to our classification for age in our study, we found no differences between young and elderly patients for the risk of PEP. We further grouped our patients according to age decades and found that subjects aged between 21 and 30 years had the highest PEP risk while the difference was not significant.

PEP which is a complication related to the endoscopist’s skills and technique, is still an important issue due to its reported occurrence in 2–9% of the cases in the prospective series. PEP incidence increases up to 30% in some series due to different inclusion criteria. According to Clarke et al., pancreatitis after ERCP occurs in %5 (1/21) of the patients aged 85 above, which is similar to rate among the younger patients^12^. Based on reports of Koklu et al., there was a higher frequency of pancreatitis in the younger subjects (age ≤ 69 years [2.5%] vs. ≥ 70 years [1.0%])^13^. In our study 21 patients (4.3%) aged 65 years and over suffered from PEP. In the ERCP study reported by Rodriguez-Gonzalez et al. there was no PEP among the patients aged above 90 years^14^. In our study, the number of patients aged above 90 years were 17, and pancreatitis was none. Possible reasons for the decreased ratio of PEP over 90 years old is probably age related pancreatic changes. Decreased enzyme secretion of pancreas, development of pancreatic fibrosis and atrophy in the elderly might decrease the risk of pancreatitis (17).

ERCP has very serious complications other than PEP. Although higher rate of bleeding is expected in elderly patients due to the frequent use of NSAID and antithrombotic drugs there was no significant difference in the rates of ERCP-related perforation, cholangitis, and bleeding between two groups in our study. The most prevalent complication of ERCP (4.3%) was pancreatitis. We found no increase in the post ERCP pancreatitis risk with age. Retrospective design of our study is one of the limitations. Deans et al. have studied 958 patients (677 were the age of 65 years and older, 281 were younger than 65 years of age) prospectively. In their study, the patients have been divided into age groups as younger than 35 years, 35–44 years, 45–54 years and 55–64 years. There has not been shown a statistically significant difference in post ERCP pancreatitis between these age groups, and the results were similar to our study. Nevertheless, in a study by Dean et al. with the regression analysis, the risk of all post ERCP complications increases by 2.6 fold in the group younger than 35 years of age (OR: 2.6%95 CI 0.65–11.2)^15^. Our study divided the age groups into decades, and we showed that the 20–30 age-decade group has the highest risk in terms of PEP.

Women have a higher risk of developing PEP. Female gender has been delineated as an independent risk factor of PEP by multivariate analysis in the largest retrospective and^16,17^, prospective trials^5–7^ and in a metanalysis (odds ratio (OR) 2.23: confidence interval of 95% (CI) (1.75, 2.84). Our study suggests that the most important post ERCP pancreatitis risk factor in the elderly is female gender (OR = 8.1, 95% CI 1.7–37.7, *p* = 0.001). Almost exclusively, patients who had SOD are women, which may explain the increased susceptibility of the female gender to PEP development. In a multicenter study Freeman et al. demonstrated that 19.1% (2.7% severe) of patients who had suspected SOD had PEP as compared to 3.6% (0.05% severe) of the patients who had indications other than SOD^7,9,18^. In the literature, reported PEP rates range between 10 and 30% in patients with suspected SOD^7,9,19^. Our study suggests that the elderly with post ERCP pancreatitis had more prevalent ERCP diagnosis of SOD than the patients without pancreatitis, but the difference did not reach significance. (14.3% vs. 3.8%; *p* = 0.059).

Variations in anatomy such as periampullary diverticulum and pancreas divisium have all been implicated as possible variables for PEP, but existing data is inconclusive. Sökmen et al. reported that the periampullary diverticula are common, especially in older and female patients, however do not significantly increase the risk of some complications such as perforation and bleeding. PEP is the major post ERCP complication in patients suffering from periampullary diverticula. The difficult cannulation due to JPD might be leading cause of PEP^20^. However, Katsinelos et al. stated that periampullary diverticula did not cause technical difficulties at ERCP and the risk of complications was not enhanced in elderly patients (24). In our study we found that risk of PEP increases slightly with SOD and JPD in elderly.

We found no significant difference between severity of PEP in young and elderly group. When we compare patients according to age decades still the severity of PEP was similar between groups. However we observed highest APACHE II and Ranson scores in 7th decade. The three of patients at that group had complication of bleeding that explain higher scores.

The biggest limitation of our study is its retrospective design. Second limitation is we did not included ERCP procedures performed at outpatient settings. On the other hand our clinic is one of the tertiary referral center and has large spectrum of patients. In conclusions: This is one of the largest series that evaluates the post ERCP pancreatitis risk factors and rate among the elderly. According to our findings, age does not increase PEP. The most important PEP risk factor among the elderly is female gender, while the risk increases slightly with SOD and JPD. The results of that study suggest that advanced age should not hinder endoscopist for performing ERCP procedure if indication present.
