# Author Correction: Prognosis and risk factors of ERCP pancreatitis in elderly

**DOI:** 10.1038/s41598-021-97721-6

**Published:** 2021-09-01

**Authors:** Erhan Ergin, Nevin Oruç, Galip Ersöz, Oktay Tekeşin, Ömer Özütemiz

**Affiliations:** Gastroenterology Department, Faculty of Medicine, Ege University, Bornova, Izmir, Turkey

Correction to: *Scientific Reports* 10.1038/s41598-021-95484-8, published online 05 August 2021

The original version of this Article contained an error in Table 3, where the “Complications” for “Syrén et al. (2019)^36^” was given in Turkish.

“Belirtilmemiş”

now reads:

“Unspecified”

The original Article has been corrected.

